# Detection of *Mycobacterium avium* Subspecies *paratuberculosis* (MAP) Microorganisms Using Antigenic MAP Cell Envelope Proteins

**DOI:** 10.3389/fvets.2021.615029

**Published:** 2021-02-03

**Authors:** Shanmugasundaram Karuppusamy, Lucy Mutharia, David Kelton, Brandon Plattner, Sanjay Mallikarjunappa, Niel Karrow, Gordon Kirby

**Affiliations:** ^1^Department of Biomedical Sciences, Ontario Veterinary College, University of Guelph, Guelph, ON, Canada; ^2^Department of Molecular and Cellular Biology, College of Biological Science, University of Guelph, Guelph, ON, Canada; ^3^Department of Population Medicine, Ontario Veterinary College, University of Guelph, Guelph, ON, Canada; ^4^Department of Pathobiology, Ontario Veterinary College, University of Guelph, Guelph, ON, Canada; ^5^Department of Animal Biosciences, Ontario Agricultural College, University of Guelph, Guelph, ON, Canada

**Keywords:** mycobacterium, cell envelope, ELISA, immunohistochemistry, immunomagnetic separation

## Abstract

Cell envelope proteins from *Mycobacterium avium* subspecies *paratuberculosis* (MAP) that are antigenically distinct from closely related mycobacterial species are potentially useful for Johne's Disease (JD) diagnosis. We evaluated the potential of ELISAs, based on six antigenically distinct recombinant MAP cell envelope proteins (SdhA, FadE25_2, FadE3_2, Mkl, DesA2, and hypothetical protein MAP1233) as well as an extract of MAP total cell envelope proteins, to detect antibodies against MAP in the sera of infected cattle. The sensitivity (Se) and specificity (Sp) of an ELISA based on MAP total cell envelope proteins, when analyzing 153 bovine serum samples, was 75 and 96%, respectively. Analysis of the same samples, using a commercial serum ELISA resulted in a Se of 56% and Sp of 99%. Results of ELISA analysis using plates coated with recombinant cell envelope proteins ranged from a highest Se of 94% and a lowest Sp of 79% for Sdh A to a lowest Se of 67% and a highest Sp of 95% for hypothetical protein MAP1233. Using polyclonal antibodies to MAP total cell envelope proteins, immunohistochemical analysis of intestinal and lymph node tissues from JD-positive cattle detected MAP organisms whereas antibodies to recombinant proteins did not. Finally, polyclonal antibodies to MAP total cell envelope protein and to recombinant SdhA, FadE25_2, and DesA2 proteins immunomagnetically separated MAP microorganisms spiked in PBS. These results suggest that antigenically distinct MAP cell envelope proteins and antibodies to these proteins may have potential to detect MAP infection in dairy cattle.

## Introduction

Johne's disease (JD) is a non-treatable chronic granulomatous enteritis of cattle and small ruminants caused by *Mycobacterium avium* subspecies *paratuberculosis* (MAP) (1). JD is associated with profuse diarrhea, emaciation, submandibular edema, and eventually death of infected animals due to poor nutrient absorption. JD is endemic in North America, prevalent worldwide and imposes considerable economic burden to the cattle industry due to production losses and herd replacement costs (2, 3). There are four stages in JD. In the silent stage I, infected animals are healthy without shedding of MAP in the feces (4). In stage II, the disease is subclinical and infected animals appear healthy and can intermittently shed MAP in the feces, thereby contaminating the environment and acting as a source of infection to herd-mates (4). Current laboratory tests have very limited sensitivity in the diagnosis of animals at stage I and II of infection and cattle may remain undiagnosed for several years (5). In stages III (clinical disease) and IV (advanced clinical disease), infected animals exhibit typical clinical signs of JD such as intermittent to continuous diarrhea, weight loss, and emaciation and shed large numbers of MAP in the feces (4).

Currently, JD is diagnosed by clinicians and pathologists using fecal culture, PCR, ELISA, and the identification of gross and histopathological lesions in infected tissues including the presence of acid-fast bacilli (6). Culturing MAP from infected tissues is considered to be the most accurate direct detection test for JD diagnosis (7). However, due to the low numbers of MAP in infected tissues and the disparate distribution, multiple tissue samples are necessary to isolate and culture MAP microorganisms, a process that typically takes 5–16 weeks (7). While direct visualization of MAP by acid-fast staining of intestinal smears and sections is also employed, acid-fast staining has limited sensitivity and specificity as it requires a minimum of 10^6^ MAP organisms per gram of tissue and non-specific staining of other acid-fast bacterial species occurs (8, 9). Alternatively, direct detection of MAP in infected tissue by immunohistochemistry using MAP-specific antibodies is a more accurate technique that can detect both intact and lysed MAP organisms (9).

The design of studies to assess tests for JD is problematic due to the difficulty in identifying a suitable reference standard for comparison purposes. While fecal culture is considered to be the gold standard test for identification of MAP microorganisms (7), there are several inadequacies in that the test has limited sensitivity, is time-consuming, labor-intensive, and expensive (7). Moreover, the use of chemical decontaminants reduces the viability of MAP microorganisms and affects the sensitivity of the assay (10). In addition, MAP microorganisms are often shed intermittently in the feces and the number of microorganisms shed by low and medium shedders is minimal (5, 11) and the lack of efficient methods to concentrate MAP from the samples reduces the sensitivity and specificity of MAP detection by culture. Detection of MAP DNA in the feces is also used in JD diagnosis. Isolation of high quality MAP DNA from feces is also challenging due to low numbers of MAP microorganisms in the feces and difficulty in lysing cells to extract DNA (7). In addition, the presence of PCR inhibitors in fecal matter affects the sensitivity of PCR-based identification of MAP (12). Immunomagnetic capture of MAP allows a selective concentration of the organism from other non-specific bacteria and inhibitory substances (13). Captured bacteria can then be identified by other methods such as culture, or amplification via phage display methods or PCR (10, 13).

ELISA is a commonly used test by clinicians and pathologists to diagnose JD, due to its simplicity and cost-effectiveness. In general, the sensitivity and specificity of commercial ELISA kits varies from 45 to 57% and 85 to 99%, respectively, for fecal culture-positive cases (1, 14). Part of the variations in ELISA sensitivity are due to fluctuations in the antibody titer depending on the stage of infection (15). While comparisons of different tests are questionable when data are not paired, there is variability between commercial ELISA kits with samples showing seropositivity by one and seronegativity by another (16, 17). Moreover, a recent analysis of cow serum samples from MAP-infected and uninfected animals with a commercial ELISA revealed a sensitivity of 4.5% in comparison to an ELISA using recombinant MAP1985 antigen (18). Indeed, none of the commercial ELISA kits can be used as a single test to identify early stage MAP infection in dairy cattle (19). Selection and incorporation of MAP antigens that are both specific and sensitive in an ELISA is a challenging task due to genetic similarity of MAP with other subspecies within the *M. avium* complex and sharing of antigenic epitopes with other mycobacterial and non-mycobacterial species (6). Exposure of animals to related bacterial species may generate antibodies that cross-react with MAP antigens affecting the specificity of MAP ELISA tests (20). Identification of MAP-specific antigens that could be incorporated into ELISAs might be valuable in JD diagnosis. Indeed, flow cytometry analysis has shown that antibody binding to MAP cell surface antigens is particularly sensitive and subspecies-specific (21).

While commercial ELISAs are most commonly used in the serodiagnosis of JD, test specificity is limited by the use of crude antigen preparations such as purified protein derivative, protoplasmic antigens, and lipoarabinomannan that contain epitopes expressed in other mycobacterial and non-mycobacterial species (22, 23). This can lead to false positive diagnoses of JD due to cross-reacting antibodies that are not related to MAP exposure (21). For instance, estimated specificities and sensitivities of five commercial ELISA tests for the diagnosis of JD varied from 87.4 to 99.8% and 27.8 to 44.5%, respectively, in comparison to fecal culture (14). A previous study revealed that ELISA plates with formalin-treated whole MAP organisms or cell surface proteins extracted from formalin-treated, sonicated MAP organisms produced a sensitivity and specificity of more than 95% in the serodiagnosis of JD (1). Another study used flow cyotometry to detect MAP subspecies-specific IgG antibodies against MAP cell surface antigens from cattle subclinically infected with MAP and showed that these antigens could be used in the serodiagnosis of JD (24).

We have recently identified several MAP cell envelope proteins that are antigenically distinct from genetically close species including *M. avium* subsp. *hominisuis* (MAH) and *M. smegmatis*, an environmental mycobacterium (25). In this study we investigate whether the use of extracts of MAP total cell envelope proteins or individual antigenically distinct recombinant MAP-specific cell envelope proteins in an ELISA format would improve the sensitivity and specificity of *M. phlei*-preabsorbed sera as has been demonstrated in similar studies (20, 21, 26). Thus, the objectives of this study are to assess the potential of ELISAs that incorporate six antigenically distinct MAP recombinant cell envelope proteins SdhA, FadE25_2, FadE3_2, Mkl, DesA2, and hypothetical protein MAP1233 to detect serum antibodies to MAP. In general, the functional roles of these specific cell envelope proteins are to support MAP survival and persistence during infection as we have previously discussed (25). Briefly, succinate dehydrogenase (SdhA) allows mycobacteria to adapt to hypoxic environments by maintaining ATP synthesis, Acyl coA dehydrogenase (FadE25_2 and FadE3_2) participates in b-oxidation of cholesterol producing carbon as an energy source; little is known about the Mkl gene product in MAP, however, it is involved in the acquisition of carbohydrates by *Mycobacterium tuberculosis* in host macrophages; disruption of the DesA2 gene, encoding Acyl-ACP desaturase reduces viability of pathogenic mycobacteria, and hypothetical protein MAP1233 has sequence similarity with the methyltransferase FkbM family which is associated with cell wall biogenesis and remodeling. An ELISA using an extract of MAP total cell envelope proteins was also assessed. In addition, we tested the utility of polyclonal antibodies to MAP total cell envelope proteins in the identification of MAP organisms by immunohistochemistry and by immunomagnetic separation. As such, the goal of this proof-of-concept study was to assess the accuracy of a variety of approaches using MAP cell envelope proteins, and polyclonal antibodies to these proteins, in identifying the presence of MAP microorganisms in a small population of cattle (*n* = 153).

## Materials and Methods

### Bacterial Strains, Media, and Growth Conditions

Three MAP strains (Madonna, gc86, and gd30 strains), isolated from bovine clinical cases from southern Ontario, Canada were graciously were grown in Middlebrook 7H9 broth medium at 37°C for 6–8 week as described in our previous study (25). Cultures were harvested by centrifugation at 1,000 g for 30min at 4°C and washed three times with ice-cold phosphate buffered saline (PBS) (pH 7.4). Bacterial pellets were then washed with a 0.16M NaCl solution. Subcellular fractionation of MAP was done to obtain the cell wall core and cytoplasmic membranes using lysozyme digestion, bead beating and ultracentrifugation as previously described (25).

### Recombinant Protein Antigen Purification

Six antigenic proteins were purified from the MAP cell envelope expressed in *Escherichia coli* BL21 (DE3) based on our previous study (25). Five recombinant proteins (SdhA, FadE25_2, FadE3_2, Mkl, and DesA2) were purified under native conditions with immobilized metal affinity chromatography techniques using HiTrap^TM^TALON® crude resins (GE Healthcare, Bio-Sciences AB, Uppsala, Sweden) as per manufacturer's instructions. A hypothetical protein MAP1233 was purified under denaturing conditions using HisPur^TM^ Ni-NTA (Nickel-Nitrilotriacetate) resin (Thermo Scientific, Rockford, IL, USA), as per the manufacturer's instructions. Protein purity was assessed by sodium dodecyl sulfate–polyacrylamide gel electrophoresis sodium dodecyl sulfate-polyacrylamide gel electrophoresis (SDS-PAGE) and Coomassie blue staining (27). Proteins purified under native conditions were dialyzed in PBS (10mM, pH 7.4) and 10–20% glycerol was added. Protein concentrations were quantified by bicinchoninic acid assay using a Pierce BCA Protein Assay kit (Thermo Scientific, Rockford, IL, USA), and samples were aliquoted and stored at −80°C until further use. It was not possible to solubilize the denatured hypothetical protein so the denatured form was subsequently used for ELISA experiments.

### Sample Collection

Fecal and serum samples were obtained from 153 adult milking dairy cows (ranging from 2 to 6 years of age) from three dairy herds in southwestern Ontario. Cattle had been tested for JD based on both fecal culture (FC) and commercial ELISA kits for antibodies to MAP in serum(MAP Ab Test, IDEXX Laboratories Inc., Westbrook, ME; USA) and milk (Prionics ParaChek test kit analysis of milk, Prionics, Zurich, Switzerland). Herd R1 had an average of 90 milking cows (70% Jersey and 30% Holstein) with an average herd prevalence of 20% based on 6 whole herd milk ELISA tests over 6 years. Herd R2 had an average of 38 milking cows (all Jerseys) with an average herd prevalence of 11% based on 11 whole herd milk ELISA tests over 6 years. Herd R3 had an average of 120 milking cows (all Holsteins) with an average herd prevalence of 8% based on 8 whole herd milk ELISA tests over 3 years. In Supplementary Table 1, fecal culture results revealed that 39 cows were positive and 114 were MAP-negative for MAP microorganisms. Commercial serum ELISA results revealed that 24 were positive for MAP serum antibodies and 129 were negative.

### Serum Absorption

One isolate of each of three mycobacterial species i.e., *M. avium* subsp. *hominisuis* (MAH), *M. smegmatis*, and *M. phlei* were used for serum absorption. In brief, frozen glycerol stock cultures were streaked on Middlebrook7H11 agar plates and incubated at 37°C. From these plates, single colonies for each isolate were picked and sub-cultured in 15 mL of Middlebrook7H9 broth at 37°C after incubation, 4 mL of culture from each isolate was aseptically aliquoted and sub-cultured into Middlebrook7H9 culture media (250 mL × 2 flasks/bacteria) and cultures were harvested separately by centrifugation at 3,000 g for 20 min at 4°C and washed twice with PBST. One suspension was heat-killed at 100°C for 15 min, cooled at room temperature and washed three times with PBS. The other pellets had neutral buffered formalin added (0.5% final concentrations) and was incubated at room temperature for 2 h on a rotating platform followed by repeated washing (3 times) with PBS. Heat and formalin-killed bacterial pellets were suspended in PBS, pooled and stored at 4°C until further processing. Serum samples were diluted (1:100) in 2% bovine serum albumin (BSA) in PBS with 0.5% Tween 20 containing killed MAH, *M. smegmatis*, and *M. phlei* (10% v/v) and were then incubated at 4°C overnight on a rotating platform. Absorbed serum samples were centrifuged at 13,000 g for 20 min at 4° C. Supernatants were then transferred into new microcentrifuge tubes and stored at −20°C until further processing.

### Validation of ELISA With MAP Cell Envelope Proteins and Recombinant Proteins

The checkerboard titration method was used to optimize the indirect ELISA components such as coating buffer, blocking buffer, antigen concentrations, primary antibody dilutions, and conjugate dilutions as previously described (28). To optimize the primary antibody dilutions, a total of 10 bovine serum samples that were JD test-positive by fecal culture and IDEXX serum ELISA were pooled and served as a positive control. Similarly, bovine serum samples (*n* = 10) that were JD test-negative by fecal culture and IDEXX serum ELISA were pooled and served as negative controls. After this optimization of ELISA components, subsequent ELISAs were performed with single dilutions of antigens and antibodies. A total of 153 serum samples from cows with known status for MAP based on fecal culture results were used so that relative sensitivities and specificities could be calculated in order to validate the new assay. In brief, MAP cell envelope proteins were diluted in bicarbonate coating buffer to a final concentration of 250 ng/mL and 100 μL of diluted antigen was added to each well of the 96-well microtiter plates. Plates were incubated at 4°C on a shaker (45 rpm) overnight and washed three times with PBS using an automated plate washer. Wells were blocked with 2% BSA (IgG-free) (Santa Cruz Biotechnology, Dallas, USA) in PBS, incubated for 2 h at room temperature on a shaker (85 rpm) and washed three times with PBS. Absorbed serum samples were diluted to 1:1,000 for MAP total cell envelope proteins and 1:500 for recombinant proteins in 2% BSA in PBS with 0.5% Tween 20 and each sample was added into duplicate wells. For non-absorbed serum, samples were diluted 1:1,000 in 2% BSA in PBS with 0.5% Tween 20 and each sample was added into duplicate wells. The remaining procedures were common for both the absorbed and non-absorbed samples. Plates were incubated at room temperature for 2 h on a shaker (85 rpm) and washed 6 times with PBST. Wells were then incubated with horse radish peroxidase (HRP)-linked conjugate antibody (affinity-purified rabbit anti-bovine IgG) (Jackson Immunoresearch Laboratories Inc., West Gove, PA), diluted (1:7,500) in 2% BSA in PBS with 0.5% Tween 20 for 2 h at room temperature on a shaker and washed six times with PBST. Each well-received 100 μL of highly sensitive 3,3′,5,5′-tetramethylbenzidine (TMB) substrate (Bio legend, USA), incubated for 20 min at room temperature and reactions were stopped with 100 μL of 2 N H_2_SO_4_. Readings were measured at OD_450_ using a microtiter plate reader. Experiments were repeated twice to test repeatability and reproducibility.

### Statistical Analysis

Fecal culture results were chosen as the gold standard of JD diagnosis in order to compare serum samples and to calculate the sensitivity and specificity of the ELISAs. Sensitivity and specificity of the MAP cell envelope protein ELISA and five recombinant proteins ELISAs including confidence intervals of 95% (CI-95%) were calculated from MAP-positive and -negative serum samples. The ability of the tested antigens to discriminate between MAP test-positive and test-negative animals was assessed by plotting the area under the receiver operating characteristic curve (AUC_ROC_) using MedCalc 10.3.0.0 statistical software (MedCalc®, Mariakerke, Belgium). Sensitivities and specificities were estimated based on maximum Youden index J. The influence of serum absorption on specificity of the MAP cell envelope protein ELISA was assessed using the McNemar test which accounts for paired data.

### Generation of Polyclonal Antibodies to MAP Total and Recombinant Cell Envelope Proteins

Total MAP cell envelope proteins from the three MAP isolates (Madonna, gc86, and gd30) were extracted as described above. Protein extracts were dialyzed against descending concentrations of urea, thiourea and 3-[(3-cholamidopropyl) dimethylammonio]-1-propane**s**ulfonate (CHAPS) buffers with a final dialysis with 10 mM PBS (pH 7.2). Following collection of pre-immune serum samples, three female adult Sprague-Dawley rats were immunized intramuscularly with emulsions of MAP total cell envelope proteins (150 μg/rat) mixed with equal volumes of TiterMax gold adjuvant (Sigma-Aldrich). Polyclonal antibodies were previously generated against only three recombinant proteins (SdhA, FadE25_2, and DesA2) due to issues with folding of the other proteins and the specificity of these antibodies to the respective proteins has been described (25).

### Immunoblot Analysis of Polyclonal Antibodies Generated to MAP Total Cell Envelope Proteins

For immunoblot analysis of the specificity of rat anti-MAP polyclonal antibodies, 25 μg of cell envelope proteins from MAP, MAH, and MS were electrophoretically transferred to nitrocellulose membranes. Non-specific sites were blocked with 5% skim milk in TBST and membranes were incubated with serum (1:6,000 in 2% BSA in TBST) from rats immunized with MAP total cell envelope proteins, as described above. Membranes were washed with TBST and incubated with anti-rat HRP-linked conjugate (1:2,000 dilution in 5% skim milk in TBST) for 1 h at room temperature and then probed with Clarity^TM^ Western ECL substrate (Bio-Rad Laboratories, Inc., USA).

### Immunohistochemistry (IHC) and Immunofluorescence (IF)

IHC was performed as previously described (29) using intestinal tissues and lymph nodes from adult cattle naturally infected with MAP. For negative controls, intestinal tissues and lymph nodes were obtained from calves not previously exposed to MAP (kindly provided by Dr. Brandon Plattner, Department of Pathobiology, University of Guelph). Tissue sections (5 μm) were prepared and antigens were retrieved in sodium citrate buffer at 95°C for 20 min in a water bath and allowed to cool at room temperature for 30 min. Sections were repeatedly washed with distilled water and endogenous peroxidase activity was blocked with 3% hydrogen peroxide in distilled water for 30 min at RT. Non-specific sites were blocked with goat serum for 1 h in a humidified chamber at RT, washed with Tris-buffered saline Tween-20 and incubated with rat polyclonal antibodies (1:25) against each of the three recombinant proteins (SdhA, FadE25_2, and DesA2) or polyclonal antibodies to the MAP total cell envelope proteins diluted to 1:50 in 1% BSA in TBST overnight at 4°C in a humidified chamber. Sections were then incubated with anti-rat-HRP-linked conjugate (Cell Signaling Technology, Inc., Danvers, MA, USA) diluted 1:50 in 5% skim milk in TBST and incubated for 1 h at room temperature in a humidified chamber. Tissue sections were then washed and incubated with 200 μL of ImmPACTNovaRed peroxidase substrate (Vector Lab, Burlingame, CA, USA) in the dark for 5–20 min. Slides were washed with distilled water and counter-stained with Harris' haematoxylin solution and mounted with cover slips. Slides were examined under a light microscope for the presence of antigen antibody reactions. For immunofluorescence experiments, tissue sections were processed in a manner similar to that of IHC, except endogenous inactivation of peroxidases and secondary antibodies were labeled with fluorescein isothiocyanate and diluted 1:500 in 5% skim milk in TBST. Slides were then mounted with ProLong Gold Antifade Mountant (Invitrogen, Eugene, OR, USA) as per the manufacturer's instructions.

### Immunomagnetic Separation of MAP

Magnetic protein G Dynabeads (Thermo Fisher Scientific, Mississauga, ON, Canada) were aliquoted (10 μL/tube) into 1.5 mL microcentrifuge tubes and washed with PBST (0.1%, pH 7.5), suspended in 200 μL of PBST and loaded with either 3 μg of purified rat anti-SdhA, anti-FadE25_2, and anti-DesA2 polyclonal antibodies per tube or 5 μL of rat anti-MAP (total cell envelope protein) polyclonal antibodies per tube. Tubes were incubated overnight at 4°C with gentle mixing and then placed on a magnetic stand where unbound antibodies were removed and beads were washed twice with PBST (0.1% Tween 20, pH 7.4). MAP cultures were harvested by centrifugation at 6,500 g for 10min at room temperature followed by three washing steps with PBST and bacterial pellets were re-suspended in PBST. MAP organisms were then passed through 25-gauge needles to break the bacterial clumps as previously described (30). MAP organisms were quantified by measuring optical density at 600 nm with an optical density of 0.6–0.9 at 600 nm considered to be equivalent to approximately 10^8^CFU of MAP organisms per mL as previously described (31). For exact numbers, optical density-adjusted MAP organisms (OD_600_ 0.6) were serially diluted from 10^8^ to 10^1^ in 1mL of PBST and 100 μL from each dilution was plated on Middlebrook 7H11 agar plates supplemented with mycobactin J and on Middlebrook Oleic Albumin Dextrose Catalase enrichment agar medium and plates were incubated at 37°C.

For the immunomagnetic (IM) separation, a volume of 100 μL from each dilution or from a suspension of MAP organisms was mixed with 10 μL of antibody-bound protein G beads and incubated at room temperature for 1 h with gentle mixing. For negative controls, beads were coated with polyclonal antibodies to unrelated proteins (i.e., anti-alpha-1 acid glycoprotein or anti-cytochrome P450 2A5) and incubated with MAP (10^7^ CFU) bacteria. Beads were then washed 3 times with PBST buffer in a magnetic separator to remove unbound bacteria. Immunomagnetically separated MAP was then suspended in 50 μL of sterile PBS stored at 4°C until further use.

### PCR Assay With Immunomagnetically Separated MAP

To test whether IM separation of MAP was successful, a PCR assay was performed using DNA templates prepared from IM-separated MAP using MAP species-specific (*IS900*) primers previously described (32). In brief, 10 μL of IM-separated MAP bound to beads in PBS from previous steps were transferred into new 1.5mL microcentrifuge tubes, placed on a magnetic stand and the liquid removed carefully leaving the beads remaining in the tubes. IM-separated MAP bacteria were re-suspended in 20 μL of 10mM Tris EDTA (pH 7.6), heated at 95°C in a thermal cycler for 30min and cooled on ice for 5min. For positive controls, 25 μL of MAP culture was boiled in 10mM Tris EDTA (pH 7.6) for 10min, then cooled on ice, followed by quick high-speed centrifugation. A 4 μL aliquot of these suspensions was used as the DNA template for PCR and amplification was carried out as per the cycling conditions previously described (32). PCR-amplified products were then visualized on 2% agarose gels.

### Confirmation of MAP Attachment to IM Beads by Culture

The efficiency of MAP recovery from IM-separated beads was assessed by bacterial culture by mixing a 10 μL volume of beads from each dilution of MAP organisms with 90 μL of sterile DNA grade water, plated on Middlebrook 7H11 agar plates supplemented with mycobactin J and OADC medium and plates were incubated at 37°C for 6–8 weeks. Colonies were counted and CFU were calculated as per standard procedures.

## Results

### Sensitivity and Specificity of MAP Total Cell Envelope Protein ELISA

Results of preliminary absorbance experiments to determine optimal conditions for the ELISA coated with extracts of MAP total cell envelope proteins revealed that blocking with 2% BSA (IgG-free) in PBS produced the least amount of background and therefore was used for antibody dilutions. Based on data from checkerboard titrations the following conditions were established: 25 ng of protein/well, dilutions of 1:1,000 for serum and 1:7,500 for the HRP-conjugated secondary antibodies.

Validation of the ELISA was undertaken with serum samples from cattle with associated fecal culture (FC) results, 39 of which were FC test-positive and 114 that were test-negative. Our ELISA results were compared to FC as a gold standard in order to calculate the sensitivities and specificities determined at different cut-off points. At a cut-off value of 0.611 (OD_450_), the sensitivity and specificity of the MAP total cell envelope protein ELISA without serum absorption was 72% (95% CI, 54.8–85.8) and 90% (95%CI, 83.4–95.1), respectively (Table 1). The calculated area under the ROC curve was 0.808 (95%CI, 0.735–0.867, *p* < 0.0001; Supplementary Figure 1).

**Table 1:** Calculated sensitivities, specificities, and ROC_(AUC)_ for the *Mycobacterium avium* subsp. *paratuberculosis* total cell envelope protein and IDEXX serum ELISAs.

**ELISA type**	**Serum absorption**	**Se%**	**95% CI**	**Sp%**	**95% CI**	**ROC_**(AUC)**_**	**95% CI**	**Youden index J**	**Cut-off**
MAP ENV ELISA	No	72.22	54.8–85.8	90.35	83.4–95.1	0.808	0.735–0.867	0.626	>0.611
MAP ENV ELISA	Yes	75.00	57.8–87.9	95.61	90.1–98.6	0.896	0.836–0.940	0.706	>0.384
IDEXX Serum ELISA	Yes	55.56	38.1–72.1	99.12	95.2–100	0.833	0.766–0.888	0.669	0.600

We included a serum absorption step with MAH and *M. smegmatis* in addition to the routine serum absorption that was performed with *M. phlei* in order to improve the specificity of the ELISAs. Using a cut-off value of 0.384 (OD_450_), the sensitivity and specificity of the MAP total cell envelope protein ELISA was 75 and 95.61%, respectively after absorption (Table 1). The calculated area under the ROC curve was 0.90 (Table 1 and Supplementary Figure 1). Statistical analysis revealed that serum absorption significantly improved the specificity of the ELISAs compared to non-absorption (one-tailed *p* < 0.035, exact test) with an Odds Ratio estimate of 7 and one-sided 95% CI >1.125, however, sensitivity was not improved. Thus, it is estimated that non-absorbed ELISA has 7 times the odds of giving false positives than does absorption.

In order to compare the efficiency of the MAP total cell envelope protein ELISA to the IDEXX ELISA, results were compared with FC as a gold standard. In relation to FC, IDEXX ELISA results, as per the cut-off value given by the manufacturer's recommendations, are also shown in Table 1. Of the 153 serum samples, 24 were test-positive with the IDEXX ELISA. Of these 24 animals, 23 were positive for MAP by fecal culture and one was negative. The calculated area under the ROC curve for the IDEXX ELISA was 0.833 compared to 0.896 for the MAP total cell envelope protein ELISA with serum absorption (Table 1). Comparison of ROC_(AUC)_ for the three ELISAs also revealed that the MAP total cell envelope protein ELISA using absorbed serum samples had the highest ROC_(AUC)_ value (Table 1 and Supplementary Figure 1).

### Sensitivity and Specificity of ELISAs With Recombinant Proteins

Based on preliminary absorbance values and checkerboard titrations, similar conditions were used for ELISAs with recombinant proteins except that a serum dilution of 1:500 was chosen. For all six recombinant protein antigens, OD_450_ values for MAP FC test-negative animals (*n* = 114) were less than for MAP FC test-positive animals (*n* = 39; Table 2). ROC_AUC_ analyses were performed to measure the discriminatory power of the MAP recombinant protein ELISA assay to differentiate positive and negative animals. ROC_(AUC)_ was above 0.7 for all recombinant protein antigens used in this study (Supplementary Figures 2A–F). SdhA had the highest ROC_(AUC)_ of 0.921 (95% CI 0.851–0.965) and FadE3_2 had the lowest ROC_(AUC)_ of 0.787 (95% CI 0.713–0.849) (Table 2). Youden index J analysis was performed to measure the trade-off between sensitivity and specificity at different cut-off values in order to assess the performance of the ELISAs with recombinant MAP proteins. ELISA results for SdhA protein revealed the highest Youden index J of 0.735 and FadE3_2 showed the lowest value of 0.522. Sensitivities and specificities at the selected cut-off points for individual recombinant proteins are also shown in Table 2. SdhA protein resulted in the highest sensitivity of 94% (95% CI, 80.3–99.3) and the lowest specificity of 79 (95% CI, 67.9–88.3) at a selected cut-off criterion of >0.483. Hypothetical protein MAP1233 produced the lowest sensitivity of 67% (95% CI, 49.0–81.4) and highest specificity of 95% (95% CI, 87.6–98.2) at a selected cut-off criterion of >0.543. Overall, ELISA results for the six recombinant proteins showed higher sensitivity and lower specificity than the IDEXX ELISA results.

**Table 2:** Calculated sensitivities and specificities at the selected cut-off points, ROC_AUC_, and Youden index J for the ELISAs coated with recombinant protein antigens.

**Accession ID**	**Protein name**	**Gene name**	**Mean OD**_**450**_ **values**		**Se%**	**95% CI**	**Sp%**	**95% CI**	**ROC_**(AUC)**_**	**95% CI**	**Youden index J**	**Cut-off**
			**FC –ve**	**FC +ve**								
R4MT83_MYCPC	Succinate dehydrogenase iron sulfurddharanii subunit	*SdhA*	0.400	0.711	94.1	80.3–99.3	79.41	67.9–88.3	0.921	0.851–0.965	0.735	>0.483
R4NF35_MYCPC	Acyl-CoA dehydrogenase	*FadE25_2*	0.383	0.630	70.6	52.5–84.9	89.66	82.6–94.5	0.845	0.777–0.899	0.602	>0.533
R4N5P6_MYCPC	Acyl-CoA dehydrogenase	*FadE3_2*	0.330	0.573	69.4	51.9–83.7	82.76	74.6–89.1	0.787	0.713–0.849	0.522	>0.456
R4N4C8_MYCPC	Ribonucleotide-transport ATP binding protein ABC transporter	*Mkl*	0.447	0.858	83.3	67.2–93.6	89.25	81.1–94.7	0.895	0.829–0.942	0.725	>0.579
R4MZZ5_MYCPC	Hypothetical protein		0.350	0.688	66.7	49.0–81.4	94.51	87.6–98.2	0.859	0.786–0.915	0.611	>0.543
R4N4U1_MYCPC	Acyl-ACP desaturase	*DesA2*	0.368	0.672	69.4	51.9–83.7	92.17	85.7–96.4	0.861	0.796–0.912	0.616	>0.546

### Immunoblot Analysis of Rat Polyclonal Antibodies Against MAP Total Cell Envelope Proteins

**Figure 1:**
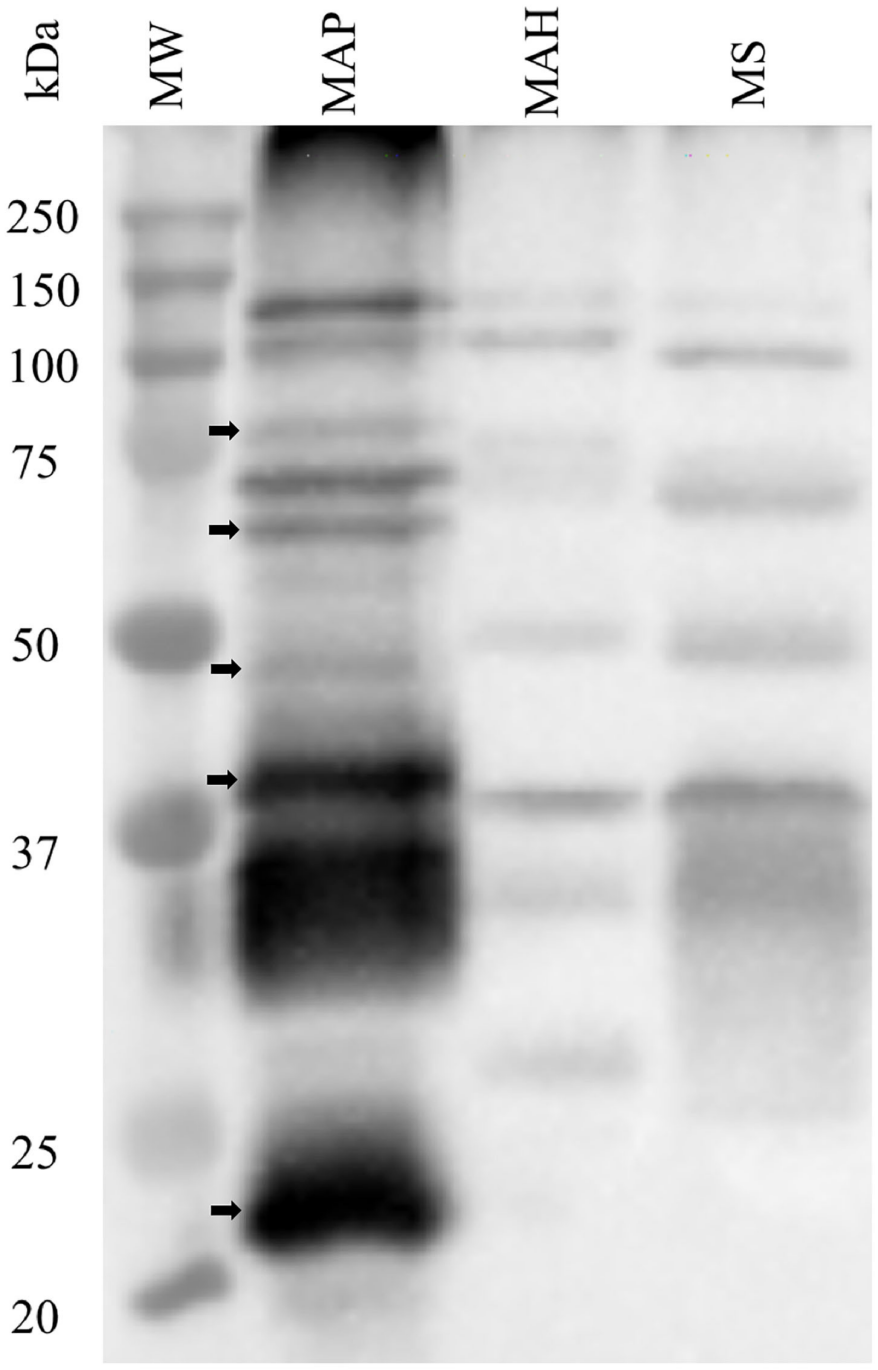
Assessment of specificity of rat polyclonal antibodies generated against cell envelope proteins from *M. avium* subsp. *paratuberculosis* (MAP). Immunoblot analysis of cell envelope protein extracts from MAP, *M. avium* subsp. *hominisuis* (MAH), and *M. smegmatis* (MS). Arrows indicate bands that are apparently specific to MAP envelope proteins.

The specificity of rat polyclonal antibodies to an extract of MAP total cell envelope proteins was assessed by immunoblot analysis of cell envelope protein extracts from MAP, MAH and *M. smegmatis* (Figure 1). The results revealed that these antibodies were strongly immunoreactive with MAP cell envelope protein extracts. However, some cross-reactivity with MAH and *M. smegmatis* cell envelope proteins was evident. Densitometric analysis of all the bands in the three lanes in Figure 1 revealed that bands in MAP, MAH and MS lanes represented 79.0, 5.9, and 15.1%, respectively, of total band density on the immunoblot.

### Immunohistochemistry and Immunofluorescence

**Figure 2:**
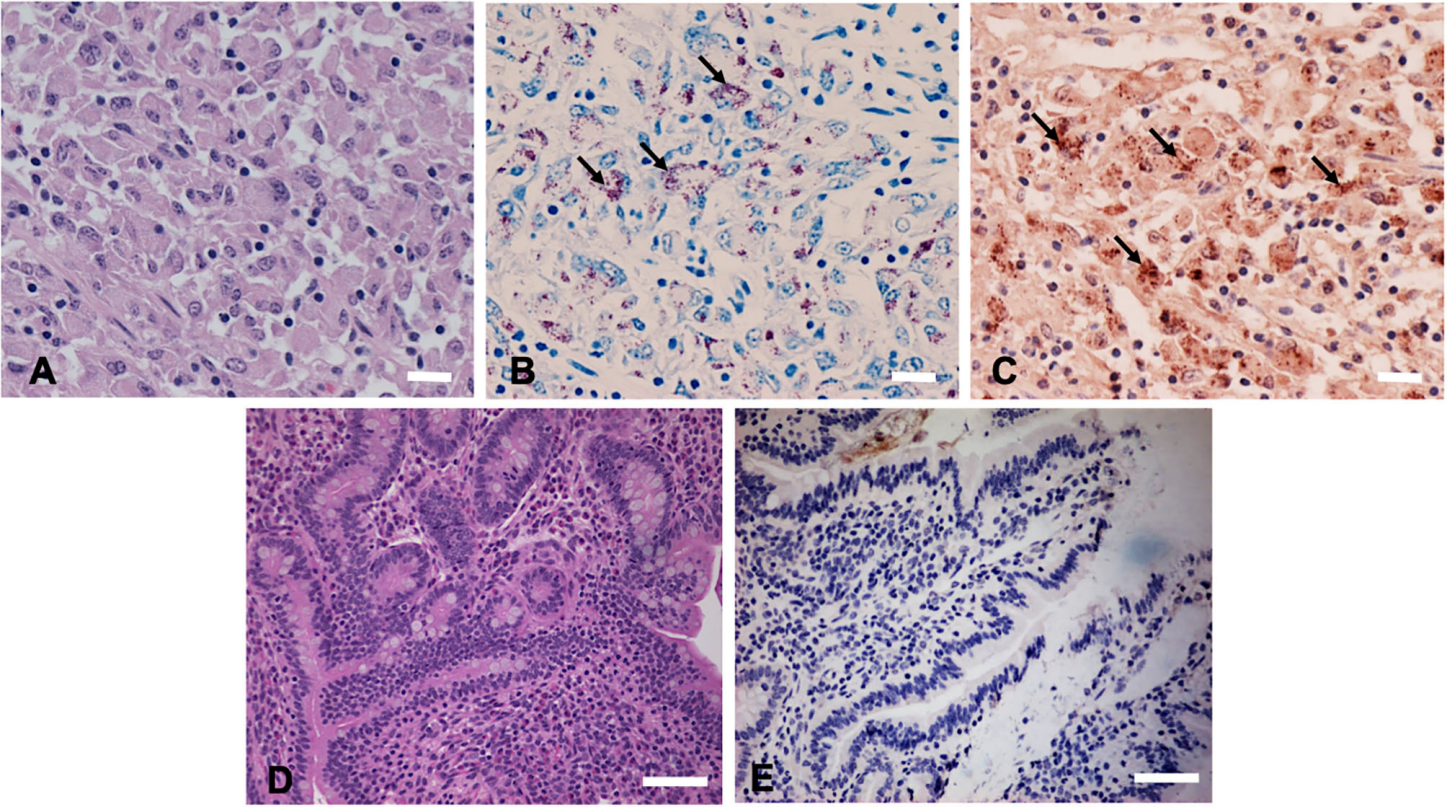
Immunohistochemical (IHC) staining of tissues sections using anti-*M. avium* subsp. *paratuberculosis* (MAP) cell envelope antibodies. **(A)** Hematoxylin and eosin stain (H&E)-stained intestinal tissue section from a MAP-infected cow showing mononuclear inflammatory cell infiltrates. Bar = 25 μm; **(B)** acid-fast staining of intestinal tissue showing the presence of MAP organisms (arrows indicating red bacilli), Bar = 25 μm; **(C)** IHC of intestinal tissue section with antibodies to MAP whole cell envelope protein extracts showing strong immunoreactivity (arrows indicating brown immunoreactivity), Bar = 25 μm; **(D)** H&E-stained intestinal tissue section from a calf not exposed to MAP, Bar = 50 μm; and **(E)** IHC of intestinal tissue section from a calf not exposed to MAP, Bar = 50 μm.

**Figure 3:**
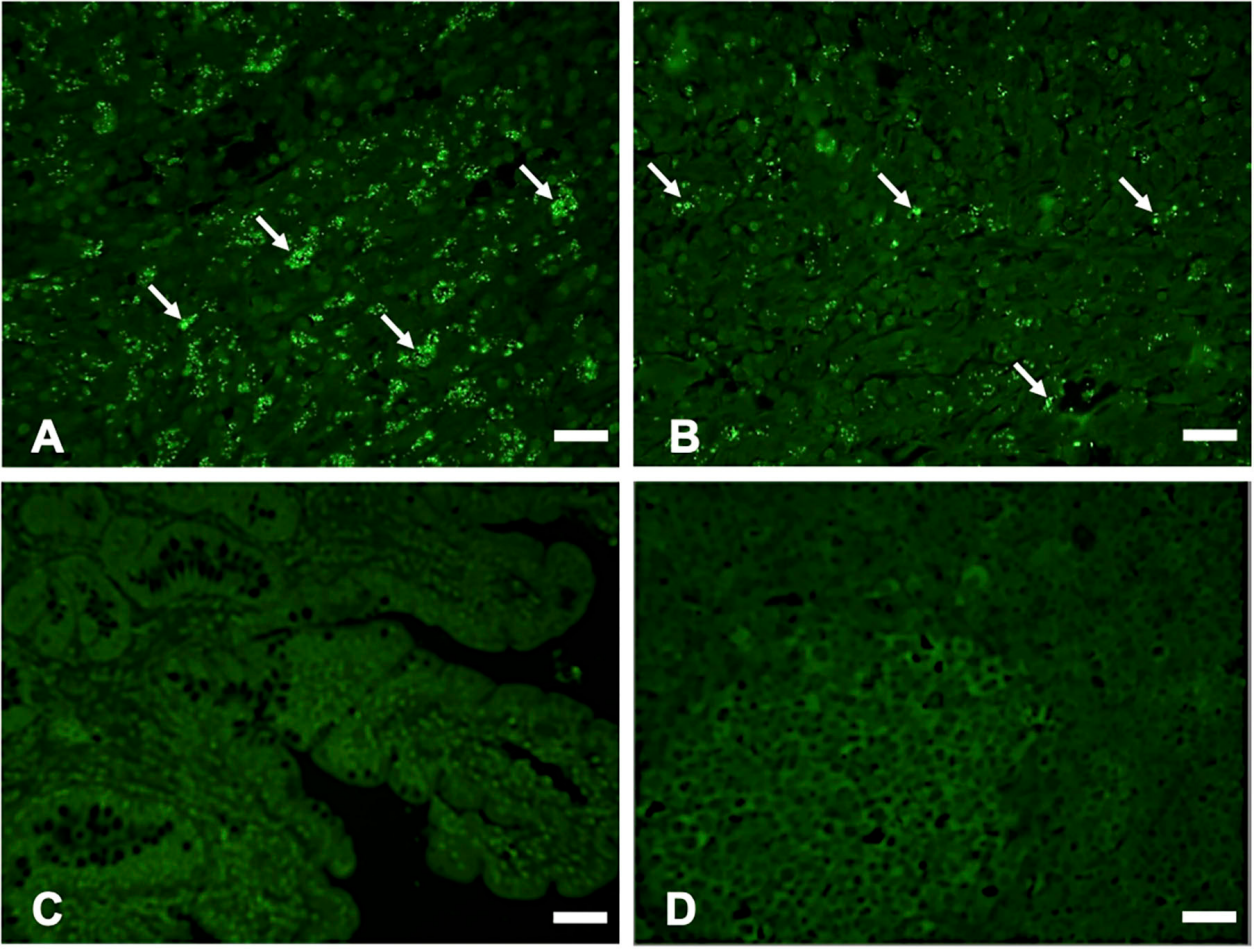
Immunofluorescence (IF) staining of tissue sections using anti-*M. avium* subsp. *paratuberculosis* (MAP) cell envelope antibodies. IF staining of intestinal tissue **(A)** and lymph node sections; **(B)** with antibodies to total MAP cell envelope protein extract showing strong immunoreactivity with MAP bacteria (arrows indicating bright green immunofluorescent spots), Bars = 25 μm; IF staining of intestinal tissue **(C)** and lymph node sections **(D)** from a calf not exposed to MAP showing lack of immunoreactivity with antibodies to total MAP cell envelope protein extract, Bars = 25 μm.

Hematoxylin and eosin (H&E) staining of formalin-fixed intestinal tissues from cattle infected with MAP demonstrated mononuclear inflammatory cell infiltrates (Figure 2A) and acid-fast staining indicated the presence of bacilli (Figure 2B). Immunohistochemistry with antibodies generated to extracts of MAP total cell envelope proteins showed strong immunoreactivity to MAP bacteria (Figure 2C). H&E staining of formalin-fixed control intestinal tissue sections from a calf not exposed to MAP showed normal histology (Figure 2D) and lacked any visible antigen-antibody reactivity (Figure 2E). Immunofluorescence using FITC-labeled antibodies identified MAP antigens in sections of intestine (Figure 3A) and lymph node (Figure 3B). There was no antigen-antibody reactivity in the control tissue sections of intestine (Figure 3C) and lymph node (Figure 3D). Finally, rat anti-SdhA, anti-FadE25_2, and anti-DesA2 polyclonal antibodies failed to identify any MAP antigens in these tissues.

### Immunomagnetic Separation (IMS) of MAP

The capturing efficiency of polyclonal antibodies to MAP total cell envelope proteins as well as to recombinant proteins SdhA, FadE25_2, and DesA2 was assessed by analysis of captured microorganisms by PCR analysis as well as by culture. PCR amplification results revealed that IMS with rat polyclonal antibodies to MAP total cell envelope proteins was most efficient, yielding the expected product size of 0.215 kbp for as low as 10^2^ CFU of MAP (Supplementary Figure 3A). These findings were confirmed by culture results. PCR analysis of IMS mRNA with rat anti-SdhA polyclonal antibodies yielded the expected product size for as low as 10^3^ CFU of MAP (Supplementary Figure 3B), whereas IMS with rat anti-FadE25_2 and DesA2 polyclonal antibodies yielded the expected product size for up to 10^5^ CFU of MAP (Supplementary Figures 3C,D). Negative control samples that included beads without antibodies or antibodies to unrelated proteins (i.e., Alpha-1 acid glycoprotein or CYP2A5) failed to produce a PCR product thereby confirming a lack of non-specific binding of antibodies. This indicates that the magnetic beads coated with polyclonal antibodies to MAP whole cell envelope protein extracts or antibodies to recombinant SdhA, FadE25_2, and DesA2 were able to bind and capture intact MAP bacteria.

## Discussion

In the first part of this study, we investigated the use of MAP total cell envelope antigens and six recombinant MAP cell envelope proteins in ELISAs developed to identify serum antibodies to MAP bacteria in naturally infected cattle. We showed that ELISAs with MAP total cell envelope proteins using absorbed serum samples significantly increased specificity. In general, ELISAs using unabsorbed serum samples are more sensitive but less specific thereby affecting test accuracy (33). In our study, serum absorption with MAH and *M. smegmatis* in addition to the traditional absorption with *M. phlei* significantly improved specificity of the assay possibly due to a reduction of cross-reacting antibodies which were generated due to environmental exposure of cattle to other mycobacterial species (34). This is in contrast to an earlier study in which two commercial ELISAs with absorbed serum samples revealed low sensitivities (13.9 and 16.6%) and specificities (95.9 and 97.1%) in comparison to fecal culture (33). In contrast, an ELISA with unabsorbed serum showed a sensitivity of 27.8% and specificity of 90% when compared to fecal culture (33). The possible reasons for the improved sensitivity in our study may be due to the use of MAP total cell envelope proteins with large numbers of MAP-specific epitopes, indigenous MAP strains, and serum absorption with MAH and *M. smegmatis*. For example, protein extracted from MAP cell surface antigens from American strains had a sensitivity of 97.1% when tested on serum samples from American origin and 21.8% when tested on serum samples from Indian origin (35, 36). Interestingly, Supplementary Table 1 shows that 6 cows (KR3-470, KR2-154, KR2-26, KR2-142, KR3-1516, and KR3-365) were negative when analyzed by the IDEXX serum ELISA and FC, but had OD_450_ values *above the cut-off value of* 0.384 with MAP cell envelope protein ELISAs. While this may represent a false positive result, the presence of MAP-specific antibodies suggests that these animals may be in the early stages of MAP infection and were not detected by fecal culture and the commercial ELISA. This may also suggest that reliance on FC as a gold standard test reduces the specificity of the MAP total cell envelope protein ELISA if indeed these cows were MAP-positive. Because intermittent shedding of MAP in the feces can limit the sensitivity of FC, tissue culture may be a more accurate gold standard method. However, confirming this contention would require postmortem sampling of MAP-infected tissues and testing fecal or serum samples from “known negative” herds, options which are not currently available for this study.

A follow-up study might be useful to determine whether these animals will subsequently test positive by the IDEXX ELISA so that the accuracy of our new MAP ELISA assays can be assessed. Similarly, some of the animals were positive by fecal culture but negative by MAP total cell envelope protein ELISA. It is possible that these animals are infected with MAP but their MAP-specific antibody levels are below the detection limit. Another possible reason may be due to passive shedding of MAP rather than actual infection (37). Discrepancy between ELISA seropositivity and fecal shedding has been reported in a longitudinal study examining correlations between serology and fecal shedding patterns (38). Nonetheless, a definitive explanation awaits re-testing of these animals to gain further epidemiological information regarding their JD status.

The specificity and sensitivity of our assay is less than that of other studies that use MAP cell surface antigens. For example, flow cytometry analysis and ethanol vortex ELISA with MAP surface antigens revealed a specificity of 96.7 and 100% and sensitivity of 95.2 and 97.4%, respectively (21, 24). However, serum samples used in these two studies were from herds with a known JD status (JD-free or -positive) and animals with a known shedding pattern (mild, moderate, and heavy fecal shedding from high prevalence herds) possibly leading to bias in the calculation of sensitivity and specificity (39). Indeed, when the ethanol vortex ELISA was tested with other serum samples (*n* = 38), results revealed that 70% of the animals were false positive for JD (35). This suggests that comparison of test accuracies between various tests for JD is not possible (39).

We also assessed an ELISA with six recombinant MAP cell envelope proteins (SdhA, FadE25_2, FadE3_2, Mkl, DesA2, and hypothetical protein MAP1233) that were identified as MAP species-specific based on our earlier two-dimensional difference gel electrophoresis comparative proteomic analysis and 2-dimensional electrophoresis immunoblot analysis (25). ELISAs with recombinant protein antigens were able to differentiate MAP-positive and -negative serum samples. This finding was in agreement with earlier studies that used similar approaches to evaluate the potential of recombinant MAP protein antigens to be used in the diagnosis of JD (40, 41). The ELISAs with SdhA and hypothetical protein MAP1233 showed the highest and lowest sensitivity of 94 and 67%, respectively. The low sensitivity of the recombinant protein ELISAs is not surprising in view of the complex nature of MAP infection. It has been shown that test using one antigen may not be sufficiently sensitive and specific during the entire course of infection and therefore future experiments with cocktails of MAP-specific recombinant protein antigens might improve the test sensitivity and allow for detection of animals at different stages of JD (42, 43).

Among the six recombinant proteins, hypothetical protein MAP1233 and DesA2 showed a high specificity of 95 and 92%, respectively. The ELISA with DesA2 recombinant protein had a ROC_(AUC)_ value of 0.84. Earlier studies with DesA2 recombinant protein ELISAs showed ROC_(AUC)_ values of 0.69 and 0.70 (44, 45). However, these studies used refolded recombinant proteins that could have altered the protein properties such as structure, orientation and antigenicity resulting in low ROC_(AUC)_ values. ELISAs with the other four recombinant proteins, SdhA, FadE25_2, FadE3_2, and Mkl, showed less specificity. In general, the specificities of ELISAs with recombinant proteins reported in this study were less than that of the commercial ELISA tests. Indeed, false positive reactions with recombinant protein-based ELISAs has been reported previously (44, 46) and considerable numbers of animals in the false positive and false negative categories are typically expected in JD diagnosis (47). In addition to the MAP-specific epitopes, it is possible that the antigens used in this study may contain other epitopes that may be present in other mycobacterial or non-mycobacterial species and environmental exposure of cattle to these microorganisms might have led to false positives. Future experiments with partial proteins or peptides as well as ELISAs coated with mixtures of different recombinant MAP cell envelope proteins could improve test specificity.

There were certain limitations to our experimental approach. In this study, we used serum samples collected from cattle from MAP-positive herds some of which were likely exposed to different levels of MAP bacteria. Moreover, a lack of true negative samples could result in a degree of bias in the calculation of sensitivity and specificity. Additional testing of true negative and true positive samples might yield a more definitive assessment of sensitivity and specificity. We acknowledge that establishing JD infection status is an important aspect of studies comparing tests for this disease. However, the dilemma is identifying a suitable reference standard test as there is no formal JD status program in Canada. The best available tests currently used for evaluation of JD in Canadian dairy herds are fecal culture (direct test) and ELISA analysis of serum antibodies to MAP (indirect test). Additional ELISA tests are available to assess antibodies in milk. However, limitations to the use of these tests associated with deficiencies in their accuracy require cautious interpretation of test results from individual cattle. For example, cattle in the early stages of infection may not have produced sufficient antibodies necessary for detection by ELISA. Moreover, MAP microorganisms may only be shed intermittently in the feces. Because only 45% of subclinically infected cattle are detected by fecal culture and <20% by serological methods, it is often necessary to repeat testing periodically to determine whether a herd has a minimal risk for MAP infection^1^.

In 2010, the USDA introduced national standards for a Voluntary Bovine Johne's Disease Control Program the purpose of which is to identify herds with low prevalence of JD (48). The program consists of a classification system with 6 levels based on annual testing with official JD tests with the highest classification levels identifying herds with 2 or more years of test negative results. However, there is no similar policy in Canada and Canadian dairy herds are typically referred to as “low prevalence” and “low risk” rather than “negative” due to limitations in existing reference tests (i.e., fecal culture and ELISA) to establish JD herd status. While the degree of infectivity can be calculated from fecal culture results as either low (<10 CFU), medium (between 10 and 50 CFU), or heavy (>50 CFU), Ontario animal diagnostic laboratories do not routinely report the number of CFUs that might reflect the level of MAP microorganisms being shed. In addition, serum samples from *M. bovis*-positive cattle could be used to further assess the level of cross-reactivity. Finally, an increase in sample size would increase the statistical power of the study.

In the second part of our study, we tested polyclonal antibodies generated to either MAP total cell envelope protein extracts, or recombinant proteins (SdhA, FadE25_2, and anti-DesA2) in order to identify MAP in tissues by IHC or immunomagnetic separation techniques. Polyclonal antibodies are routinely used in pathogen identification by immunoblot, IHC, biosensors and flow cytometry (49). Others have shown that polyclonal antibodies generated against MAP cell wall components are useful tools in the identification of MAP organisms (50). While antibodies specific to MAP are useful in the diagnosis of JD, generation of these antibodies is difficult due to the genetic similarity of MAP with other closely related mycobacterial species (51). Recently our 2-dimensional difference gel electrophoresis analysis of MAP, MAH, and *M. smegmatis* analysis showed that the MAP cell envelope proteome profile is different from genetically close relative species in the *M. avium* complex and many of the MAP-specific proteins were antigenically distinct based on 2-dimensional electrophoresis immunoblot analysis with JD-test positive cattle serum samples (25). This finding suggested that antibodies to MAP cell envelope proteins may be useful in JD diagnosis by specifically identifying MAP organisms.

IHC and IF analysis of MAP-infected intestinal and lymph node sections infected with MAP using rat polyclonal antibodies to total MAP cell envelope proteins revealed strong antigen-antibody reactions to MAP organisms. At present, there are no MAP species-specific antibodies available commercially and previous studies using commercial anti-*M. bovis* antibodies and in-house anti-MAP antibodies for IHC and IFC showed variable sensitivity when compared to various gold standard diagnostic approaches (52–54). For example, a very low sensitivity for IHC as compared to fecal culture has been reported (52). In contrast, others found that IHC was more sensitive in identifying MAP organisms in tissues sections compared to acid-fast staining (29, 55, 56). Rat polyclonal antibodies to SdhA, FadE25_2, and DesA2 were not able to identify MAP organisms in tissue sections possibly because these antigens were either inaccessible to the antibodies or were damaged by formalin fixation. Interestingly, antigens of membrane origin are more susceptible to decay in formalin fixative than cytoplasmic antigens (57). Other possible reasons include masking of epitopes, protein-protein interactions and changes in protein conformation by formalin-mediated protein cross-linking (57). Therefore, further studies with frozen tissue sections or tissues fixed in formalin for shorter periods are necessary to test the use of anti-SdhA, FadE-25_2, and DesA2 antibodies in IHC and IF.

Additionally, polyclonal antibodies from chickens (IgY) are more specific and sensitive in MAP capturing by magnetic separation (53). In experiments involving immunomagnetic separation of MAP, we found that Dyna protein G beads coated with polyclonal antibodies generated against MAP total cell envelope proteins are capable of capturing MAP organisms at concentrations as low as 10^2^ CFU. Others have used IMS-PCR based assays to identify 10^3^-10^5^ CFU of MAP (58, 59). While polyclonal antibodies to SdhA, FadE25_2, and DesA2 were able to bind and retrieve MAP organisms, the sensitivity of MAP recovery was variable. This may be because antibodies to recombinant MAP proteins target single antigens that may have reduced levels of abundance. Another possible reason is that recombinant proteins may lack the appropriate tertiary structure required for antibody recognition of native antigens on the MAP cell surface. More recently, studies found that magnetic nanoparticles coated with anti-MAP polyclonal or monoclonal antibodies were effective in the identification of MAP from clinical samples (30, 60). The advantages of immunomagnetic separation methods are that they concentrate the target bacterium from the non-specific bacterial pool and inhibitory substances. This then facilitates rapid and sensitive identification of MAP by the downstream detection tests such as PCR, culture, and acid-fast staining of MAP (13, 53, 61). Having determined the capacity of immunomagnetic separation to bind and extract MAP in PBS, future experiments will be conducted using relevant biological samples (e.g., milk or feces) that have been spiked with varying concentrations of MAP either alone or mixed with other bacterial species.

## Data Availability Statement

The original contributions presented in the study are included in the article/Supplementary Material, further inquiries can be directed to the corresponding author/s.

## Ethics Statement

This animal study was reviewed and approved by Animal Care Committee of the University of Guelph.

## Author Contributions

GK, LM, DK, BP, and NK designed the overall study. SK and SM performed the experiments. LM, DK, and BP provided samples essential to the study. SK and GK wrote the manuscript. All authors contributed to the article and approved the submitted version.

## Conflict of Interest

The authors declare that the research was conducted in the absence of any commercial or financial relationships that could be construed as a potential conflict of interest.

## Footnotes

^1^https://www.vet.cornell.edu/animal-health-diagnostic-center/testing/protocols/johnes-disease-program

## Supplementary Material

Supplementary Table 1 Individual fecal culture (FC) and ELISA analysis results for Mycobacterium avium subsp. *paratuberculosis*.

Supplementary Figure 1 Receiver Operator Characteristic (ROC) curves for *M. avium* subsp. *paratuberculosis* (MAP) total cell envelope protein ELISAs. ROC curves compare the performance of MAP total cell envelope protein ELISA with and without serum absorption and the commercial IDEXX serum ELISA. Small circles indicate Youden index J cut-off criterion for each ELISAs. A total of 153 serum samples from cows with known status for MAP based on fecal culture results were used in the ELISA.

Supplementary Figure 2 Receiver Operating Characteristic curves for ELISAs with recombinant protein antigens. **(A)** SdhA, **(B)** FadE25_2, **(C)** FadE3_2, **(D)** Mkl, **(E)** hypothetical proteinMAP1233, and **(F)** DesA2. Small circles indicate Youden index J cut-off criterion for each recombinant protein antigen. A total of 153 serum samples from cows with known status for MAP based on fecal culture results were used in the ELISA.

Supplementary Figure 3 PCR amplification of *M. avium* subsp. *paratuberculosis* (MAP) microorganisms isolated by IMS using rat anti-MAP polyclonal antibodies with MAP species-specific gene *IS900* primers; **(A)** antibodies to extract of MAP total cell envelope proteins. PCR amplification yielded the expected size of 0.215 kbp in MAP ranging from 10^8^ to 10^2^ CFU; **(B)** antibodies to SdhA: PCR amplification yielded the expected size of 0.215 kbp from 10^8^ to 10^3^ CFU of MAP; **(C)** antibodies to FadE25_2: PCR amplification yielded the expected size of 0.215 kbp from 10^8^ to 10^5^ CFU of MAP; and **(D)** antibodies to DesA2: PCR amplification yielded the expected size of 0.215 kbp from 10^8^ to 10^5^ CFU of MAP.

Supplementary Figure 4 Original image file for the immunoblot included in Figure 1. Immunoblot represents analysis of cell envelope proteins from MAP, *M. avium* subsp. *hominisuis* (MAH) and *M. smegmatis* (MS) extracted in two separate experiments (i.e., lanes 1–4 and 4–8) to confirm reproducibility. Lanes 1–4 were included in the cropped image in Figure 1.

## Abbreviations

AUC_ROC_: area under the receiver operating characteristic curve
CFU: colony forming units
FC: fecal culture
FITC: fluorescein isothiocyanate
H&E: Hematoxylin and eosin
IF: immunofluorescence
IHC: immunohistochemistry
IM: immunomagnetic
JD: Johne's Disease
MAH: *M. avium* subsp. *hominisuis*
MAP: *Mycobacterium avium* subsp. *paratuberculosis*
MS: *M. smegmatis*
Ni-NTA: Nickel-Nitrilotriacetate
OADC: oleic acid-albumin-dextrose-catalase
PBST: phosphate-buffered saline with Tween
Se: sensitivity
Sp: specificity.
