# Supplementary material for: Detection of Mycobacterium avium Subspecies paratuberculosis (MAP) Microorganisms Using Antigenic MAP Cell Envelope Proteins
Source: Front Vet Sci. 2021 Feb 3;8:615029. doi: 10.3389/fvets.2021.615029 (PMC7887298; doi:10.3389/fvets.2021.615029)
Supplement: Supplementary Table 1 — Individual fecal culture (FC) and ELISA analysis results for Mycobacterium avium subsp. paratuberculosis. [file Table_1.docx]

**Supplementary Table 1.** Individual fecal culture (FC) and ELISA analysis results for *Mycobacterium avium* subsp. *paratuberculosis*

| **Sample No.** | **Sample ID** | **FC Results** | **MAP Env Proteins ELISA OD 450** | **IDEXX Serum ELISA OD 450** | **IDEXX Serum ELISA Results** |
| --- | --- | --- | --- | --- | --- |
| 1 | KR3-129 | Neg | 0.12 | 0.02 | Neg |
| 2 | KR3-236 | Neg | 0.128 | 0 | Neg |
| 3 | KR3-371 | Neg | 0.134 | 0.02 | Neg |
| 4 | KR1-563 | Neg | 0.136 | 0.125 | Neg |
| 5 | KR3-108 | Neg | 0.139 | 0.04 | Neg |
| 6 | KR3-1034 | Neg | 0.144 | 0.02 | Neg |
| 7 | KR3-377 | Neg | 0.144 | 0.14 | Neg |
| 8 | KR3-350 | Neg | 0.145 | 0.01 | Neg |
| 9 | KR3-1763 | Neg | 0.15 | 0.04 | Neg |
| 10 | KR3-4135 | Neg | 0.151 | 0.02 | Neg |
| 11 | KR3-100 | Neg | 0.152 | 0.01 | Neg |
| 12 | KR3-179 | Pos | 0.153 | 0.08 | Neg |
| 13 | KR1-119 | Neg | 0.155 | 0.001 | Neg |
| 14 | KR3-162 | Neg | 0.157 | 0.01 | Neg |
| 15 | KR1-194 | Neg | 0.159 | 0.025 | Neg |
| 16 | KR3-1053 | Neg | 0.161 | 0.02 | Neg |
| 17 | KR3-184 | Neg | 0.161 | 0.02 | Neg |
| 18 | KR3-196(3W) | Neg | 0.162 | 0.02 | Neg |
| 19 | KR3-133 | Neg | 0.164 | 0.01 | Neg |
| 20 | KR1-160 | Neg | 0.165 | 1.395 | Pos |
| 21 | KR3-384 | Neg | 0.17 | 0.19 | Neg |
| 22 | KR2-37 | Neg | 0.172 | 0.03 | Neg |
| 23 | KR3-212 | Neg | 0.173 | 0.02 | Neg |
| 24 | KR2-824 | Neg | 0.174 | 0.04 | Neg |
| 25 | KR1-205 | Neg | 0.176 | 0.111 | Neg |
| 26 | KR3-153 | Neg | 0.176 | 0.02 | Neg |
| 27 | KR3-130 | Neg | 0.177 | 0.05 | Neg |
| 28 | KR1-218 | Neg | 0.178 | 0.01 | Neg |
| 29 | KR1-387 | Neg | 0.178 | 0.003 | Neg |
| 30 | KR3-173 | Neg | 0.182 | 0.02 | Neg |
| 31 | KR3-116 | Neg | 0.183 | 0.03 | Neg |
| 32 | KR3-226(10X) | Neg | 0.184 | 0.022 | Neg |
| 33 | KR3-187 | Neg | 0.185 | 0.01 | Neg |
| 34 | KR3-1022 | Neg | 0.189 | 0.06 | Neg |
| 35 | KR3-183 | Neg | 0.19 | 0.01 | Neg |
| 36 | KR3-1041 | Neg | 0.193 | 0.03 | Neg |
| 37 | KR2-293 | Neg | 0.193 | 0.03 | Neg |
| 38 | KR1-186 | Neg | 0.195 | 0.021 | Neg |
| 39 | KR3-202 | Neg | 0.195 | 0.03 | Neg |
| 40 | KR1-203 | Neg | 0.197 | 0.025 | Neg |
| 41 | KR3-1044 | Neg | 0.198 | 0.01 | Neg |
| 42 | KR3-205 | Neg | 0.198 | 0.03 | Neg |
| 43 | KR3-373 | Neg | 0.202 | 0.12 | Neg |
| 44 | KR1-221 | Neg | 0.204 | 0.012 | Neg |
| 45 | KR3-376 | Neg | 0.204 | 0.03 | Neg |
| 46 | KR2-15 | Neg | 0.204 | 0.02 | Neg |
| 47 | KR3-385 | Pos | 0.204 | 0.01 | Neg |
| 48 | KR3-1049 | Neg | 0.205 | 0.02 | Neg |
| 49 | KR2-42 | Neg | 0.207 | 0.02 | Neg |
| 50 | KR3-1043 | Neg | 0.208 | 0.03 | Neg |
| 51 | KR2-39 | Neg | 0.214 | 0.07 | Neg |
| 52 | KR3-126 | Neg | 0.218 | 0.03 | Neg |
| 53 | KR1-197 | Neg | 0.221 | 0.027 | Neg |
| 54 | KR3-107 | Neg | 0.222 | 0.03 | Neg |
| 55 | KR3-3123 | Neg | 0.223 | 0.02 | Neg |
| 56 | KR1-199 | Neg | 0.224 | 0.048 | Neg |
| 57 | KR3-1067 | Pos | 0.228 | 0.04 | Neg |
| 58 | KR1-201 | Neg | 0.231 | 0.022 | Neg |
| 59 | KR2-51 | Neg | 0.233 | 0.01 | Neg |
| 60 | KR3-210 | Neg | 0.234 | 0.02 | Neg |
| 61 | KR3-83 | Neg | 0.239 | 0.02 | Neg |
| 62 | R3-B | Neg | 0.241 | 0.03 | Neg |
| 63 | KR2-463 | Neg | 0.244 | 0.04 | Neg |
| 64 | KR1-207 | Neg | 0.246 | 0.017 | Neg |
| 65 | KR3-01 | Neg | 0.247 | 0.03 | Neg |
| 66 | KR2-61 | Pos | 0.248 | 0.05 | Neg |
| 67 | KR2-193 | Neg | 0.251 | 0.07 | Neg |
| 68 | KR3-1187 | Neg | 0.252 | 0.02 | Neg |
| 69 | KR2-36 | Neg | 0.252 | 0.03 | Neg |
| 70 | KR2-44 | Neg | 0.252 | 0.01 | Neg |
| 71 | KR1-232 | Neg | 0.255 | 0.025 | Neg |
| 72 | KR1-217 | Neg | 0.255 | 0.009 | Neg |
| 73 | KR3-227 | Neg | 0.257 | 0.01 | Neg |
| 74 | KR3-314 | Neg | 0.258 | 0.02 | Neg |
| 75 | KR2-404 | Neg | 0.258 | 0.02 | Neg |
| 76 | KR1-224 | Neg | 0.259 | 0.017 | Neg |
| 77 | KR3-1057(123) | Neg | 0.262 | 0.04 | Neg |
| 78 | KR1-214 | Neg | 0.263 | 0.018 | Neg |
| 79 | KR3-201 | Pos | 0.263 | 0.27 | Neg |
| 80 | KR3-229 | Neg | 0.263 | 0.03 | Neg |
| 81 | KR3-1056 | Neg | 0.264 | 0.02 | Neg |
| 82 | KR2-21 | Neg | 0.268 | 0.09 | Neg |
| 83 | KR3-143 | Neg | 0.268 | 0.02 | Neg |
| 84 | KR3-1075 | Neg | 0.27 | 0.01 | Neg |
| 85 | KR3-117 | Neg | 0.272 | 0.03 | Neg |
| 86 | KR1-216 | Neg | 0.275 | 0.029 | Neg |
| 87 | KR2-20 | Neg | 0.275 | 0.02 | Neg |
| 88 | KR2-202 | Neg | 0.28 | 0.08 | Neg |
| 89 | KR3-1098 | Neg | 0.281 | 0.05 | Neg |
| 90 | KR3-353 | Pos | 0.283 | 0.09 | Neg |
| 91 | KR2-25 | Neg | 0.285 | 0.03 | Neg |
| 92 | KR1-175 | Neg | 0.287 | 0.039 | Neg |
| 93 | KR3-375 | Neg | 0.287 | 0.03 | Neg |
| 94 | KR1-192 | Pos | 0.292 | 0.27 | Neg |
| 95 | KR1-144 | Neg | 0.295 | 0.021 | Neg |
| 96 | KR3-1042 | Neg | 0.295 | 0.03 | Neg |
| 97 | KR2-34 | Neg | 0.296 | 0.05 | Neg |
| 98 | KR3-138 | Neg | 0.296 | 0.12 | Neg |
| 99 | KR2-93 | Neg | 0.297 | 0.02 | Neg |
| 100 | KR2-171 | Neg | 0.303 | 0.03 | Neg |
| 101 | KR3-212(19W) | Neg | 0.303 | 0.05 | Neg |
| 102 | KR2-64 | Neg | 0.304 | 0.05 | Neg |
| 103 | KR3-135 | Neg | 0.304 | 0.02 | Neg |
| 104 | KR3-1051 | Pos | 0.308 | 0.03 | Neg |
| 105 | KR3-113 | Neg | 0.309 | 0.02 | Neg |
| 106 | KR3-1038 | Neg | 0.31 | 0.47 | Neg |
| 107 | KR1-183 | Neg | 0.313 | 0.081 | Neg |
| 108 | KR3-215 | Neg | 0.315 | 0.12 | Neg |
| 109 | KR3-1013 | Neg | 0.317 | 0.03 | Neg |
| 110 | KR1-77 | Neg | 0.324 | 0.486 | Neg |
| 111 | KR2-166 | Neg | 0.328 | 0.04 | Neg |
| 112 | KR2-02 | Neg | 0.345 | 0.05 | Neg |
| 113 | KR2-01 | Neg | 0.357 | 0.04 | Neg |
| 114 | KR3-1048 | Pos | 0.369 | 0.03 | Neg |
| 115 | KR1-231 | Neg | 0.371 | 0.012 | Neg |
| 116 | KR3-148 | Neg | 0.371 | 0.07 | Neg |
| 117 | KR3-97 | Neg | 0.38 | 0.02 | Neg |
| 118 | KR3-470 | **Neg** | **0.384** | **0.04** | **Neg** |
| 119 | KR3-1014 | Pos | 0.396 | 0.53 | Neg |
| 120 | KR2-154 | **Neg** | **0.406** | **0.03** | **Neg** |
| 121 | KR2-26 | **Neg** | **0.409** | **0.65** | **Neg** |
| 122 | KR3-120 | Pos | 0.436 | 0.02 | Neg |
| 123 | KR1-171 | Pos | 0.453 | 0.095 | Neg |
| 124 | KR1-141 | Pos | 0.468 | 0.31 | Neg |
| 125 | KR1-147 | Pos | 0.487 | 0.037 | Neg |
| 126 | KR2-142 | **Neg** | **0.632** | **0.11** | **Neg** |
| 127 | KR3-1516 | **Neg** | **0.743** | **0.04** | **Neg** |
| 128 | KR3-365 | **Neg** | **0.781** | **0.42** | **Neg** |
| 129 | KR3-300 | Pos | 0.782 | 0.52 | Neg |
| 130 | KR3-1027 | Pos | 0.902 | 0.82 | Pos |
| 131 | KR1-156 | Pos | 1.07 | 1.286 | Pos |
| 132 | KR3-1050 | Pos | 1.514 | 0.9 | Pos |
| 133 | KR3-1010 | Pos | 1.779 | 1.11 | Pos |
| 134 | KR1-150 | Pos | 2.087 | 1.492 | Pos |
| 135 | KR3-1009 | Pos | 2.102 | 0.17 | Neg |
| 136 | KR3-194 | Pos | 2.393 | 1.82 | Pos |
| 137 | KR1-115 | Pos | 2.569 | 1.489 | Pos |
| 138 | KR3-1033 | Pos | 2.661 | 1.75 | Pos |
| 139 | KR3-379 | Pos | 2.75 | 1.94 | Pos |
| 140 | KR3-1001 | Pos | 3.1 | 2.21 | Pos |
| 141 | KR3-383 | Pos | 3.164 | 1.46 | Pos |
| 142 | KR2-09 | Pos | 3.315 | 2.34 | Pos |
| 143 | KR2-50 | Pos | 3.343 | 2.53 | Pos |
| 144 | KR1-157 | Pos | 3.351 | 2.232 | Pos |
| 145 | KR1-150 | Pos | 3.352 | 1.492 | Pos |
| 146 | KR3-194 | Pos | 3.362 | 1.82 | Pos |
| 147 | KR3-1052 | Pos | 3.364 | 1.5 | Pos |
| 148 | KR3-1132 | Pos | 4 | 2.01 | Pos |
| 149 | KR3-1016 | Pos | 4 | 1.9 | Pos |
| 150 | KR3-1012 | Pos | 4 | 1.71 | Pos |
| 151 | KR1-157 | Pos | 4 | 2.232 | Pos |
| 152 | KR2-52 | Pos | 4 | 2.97 | Pos |
| 153 | KR3-111 | Pos | 4 | 1.71 | Pos |

Note: Samples that are negative by the IDEXX serum ELISA and FC but positive by MAP cell envelope protein ELISA are shown in bold.
