# Supplementary figures and images for: Detection of Mycobacterium avium Subspecies paratuberculosis (MAP) Microorganisms Using Antigenic MAP Cell Envelope Proteins
Source: Front Vet Sci. 2021 Feb 3;8:615029. doi: 10.3389/fvets.2021.615029 (PMC7887298; doi:10.3389/fvets.2021.615029)

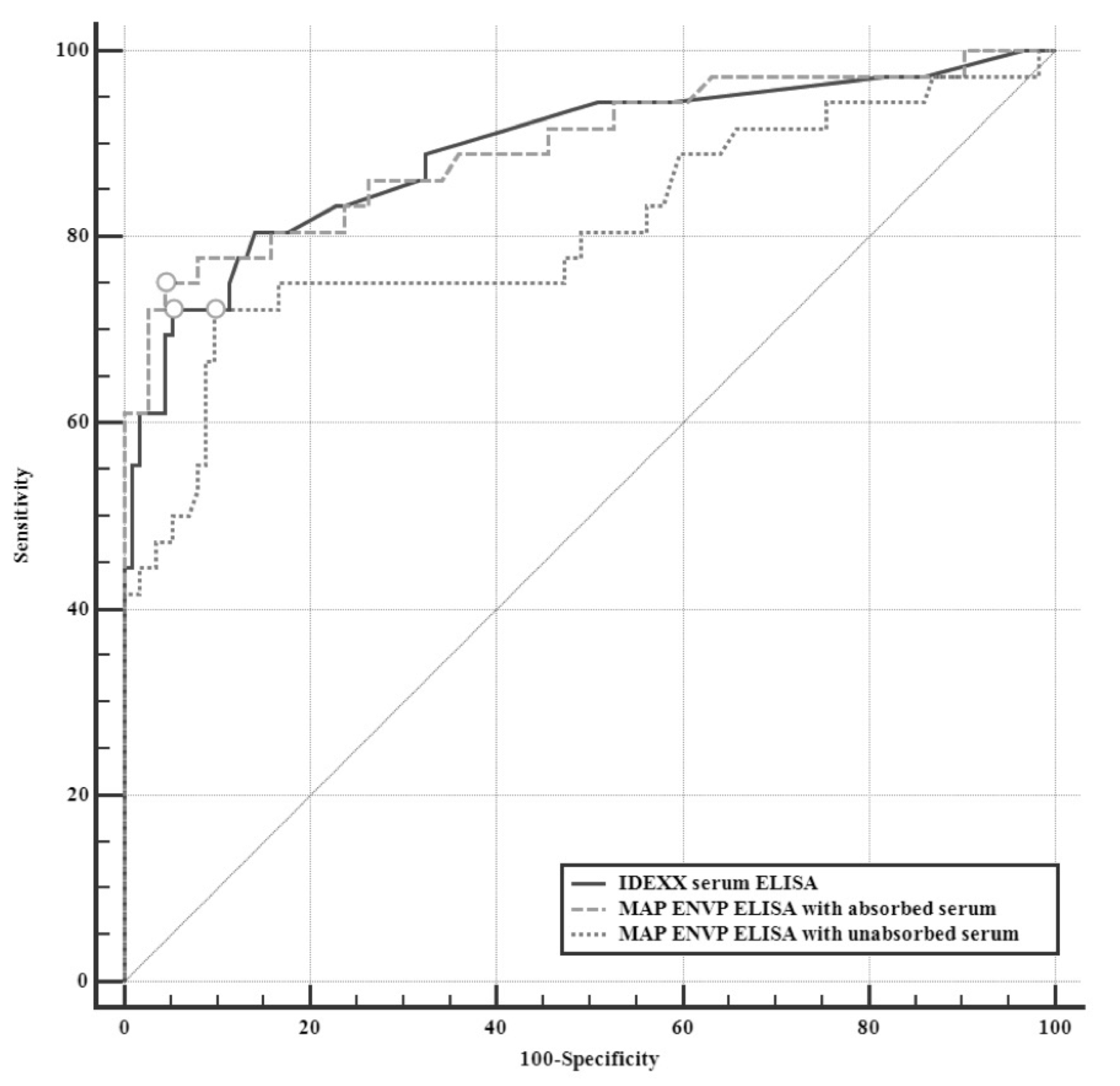

Supplement: Supplementary Figure 1 — Receiver Operator Characteristic (ROC) curves for M. avium subsp. paratuberculosis (MAP) total cell envelope protein ELISAs. ROC curves compare the performance of MAP total cell envelope protein ELISA with and without serum absorption and the commercial IDEXX serum ELISA. Small circles indicate Youden index J cut-off criterion for each ELISAs. A total of 153 serum samples from cows with known status for MAP based on fecal culture results were used in the ELISA. [file Image_1.TIFF]

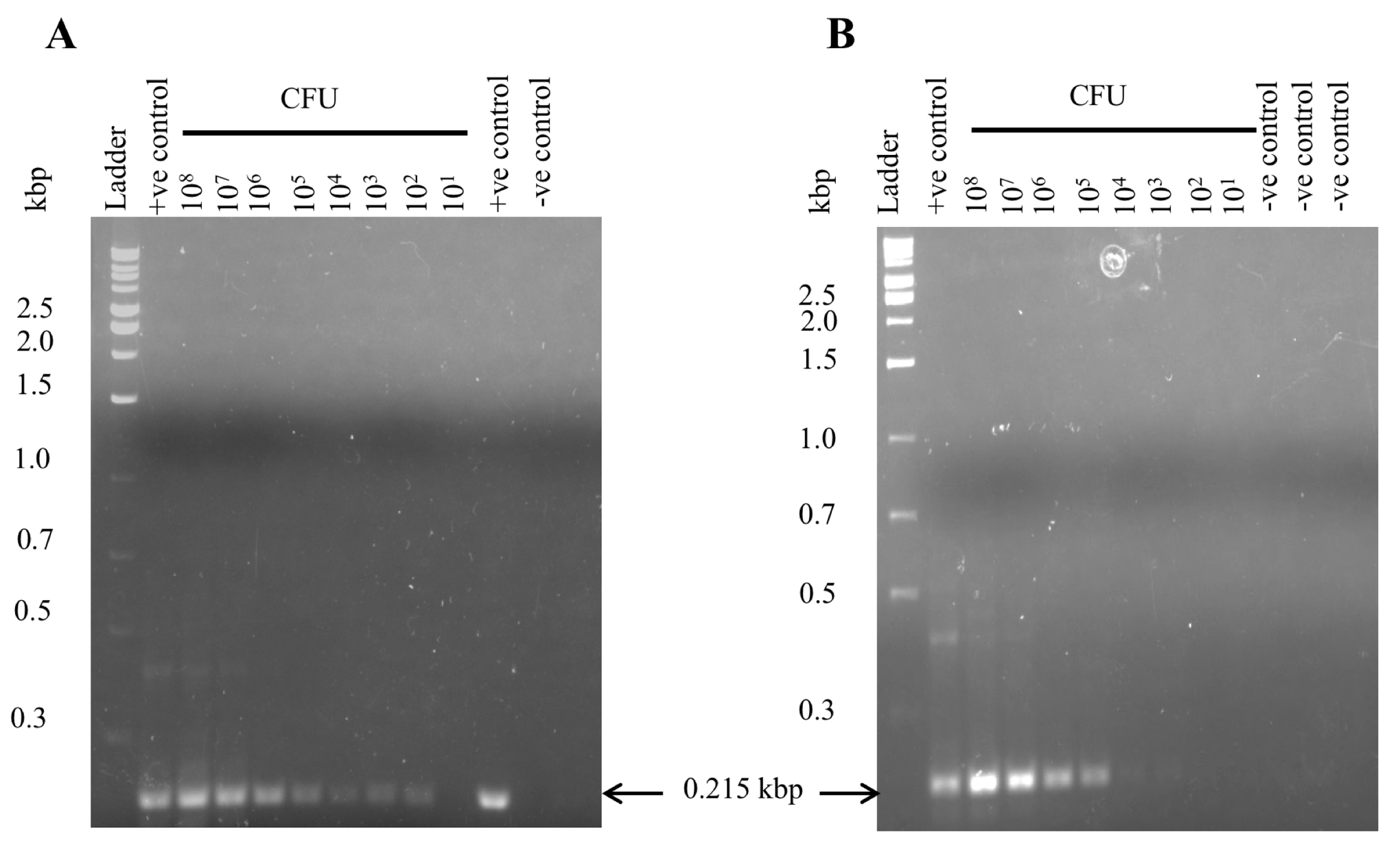

Supplement: Supplementary Figure 2 — Receiver Operating Characteristic curves for ELISAs with recombinant protein antigens. (A) SdhA, (B) FadE25_2, (C) FadE3_2, (D) Mkl, (E) hypothetical proteinMAP1233, and (F) DesA2. Small circles indicate Youden index J cut-off criterion for each recombinant protein antigen. A total of 153 serum samples from cows with known status for MAP based on fecal culture results were used in the ELISA. [file Image_2.TIFF]

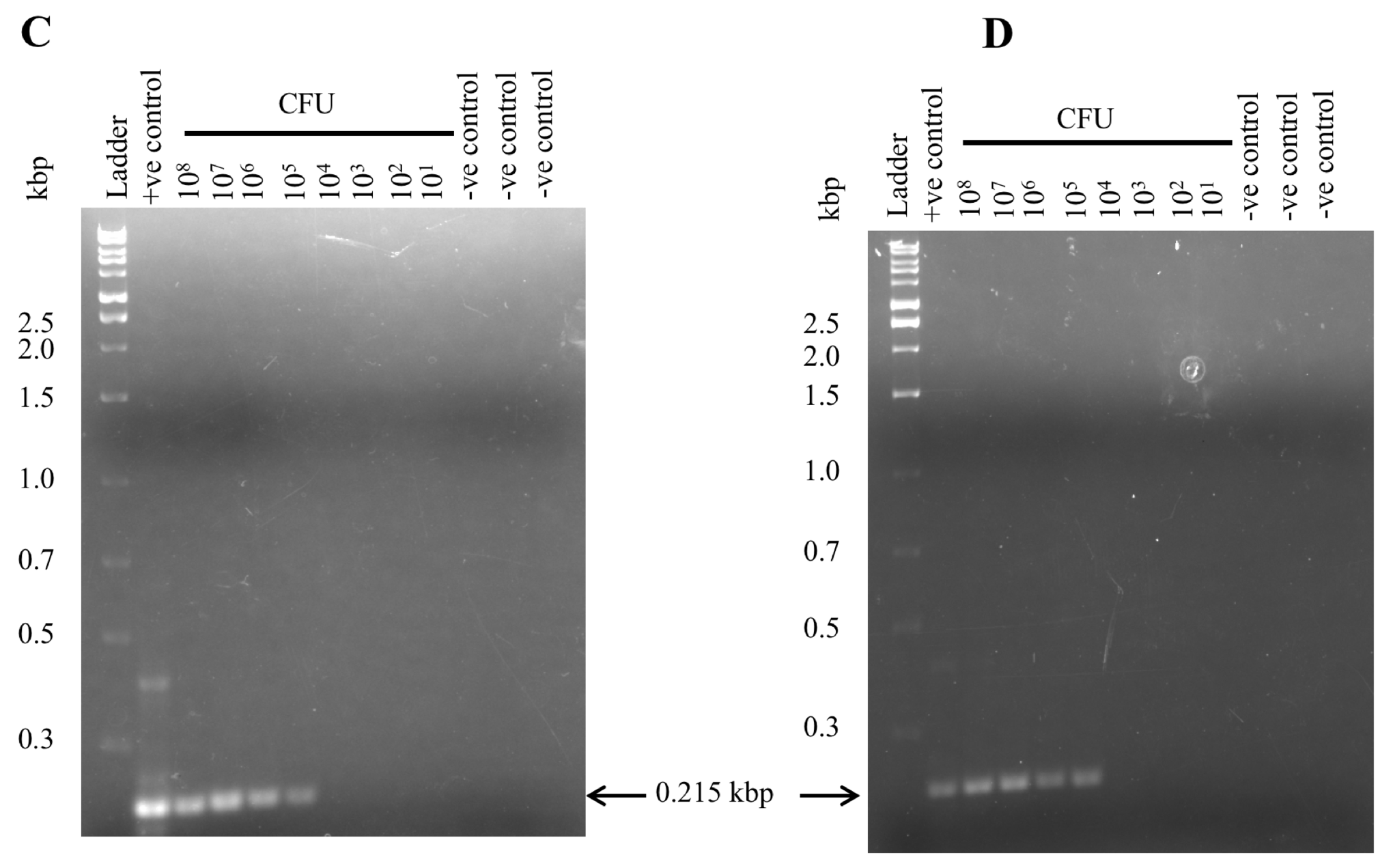

Supplement: Supplementary Figure 3 — PCR amplification of M. avium subsp. paratuberculosis (MAP) microorganisms isolated by IMS using rat anti-MAP polyclonal antibodies with MAP species-specific gene IS900 primers; (A) antibodies to extract of MAP total cell envelope proteins. PCR amplification yielded the expected size of 0.215 kbp in MAP ranging from 108 to 102 CFU; (B) antibodies to SdhA: PCR amplification yielded the expected size of 0.215 kbp from 108 to 103 CFU of MAP; (C) antibodies to FadE25_2: PCR amplification yielded the expected size of 0.215 kbp from 108 to 105 CFU of MAP; and (D) antibodies to DesA2: PCR amplification yielded the expected size of 0.215 kbp from 108 to 105 CFU of MAP. [file Image_3.TIFF]

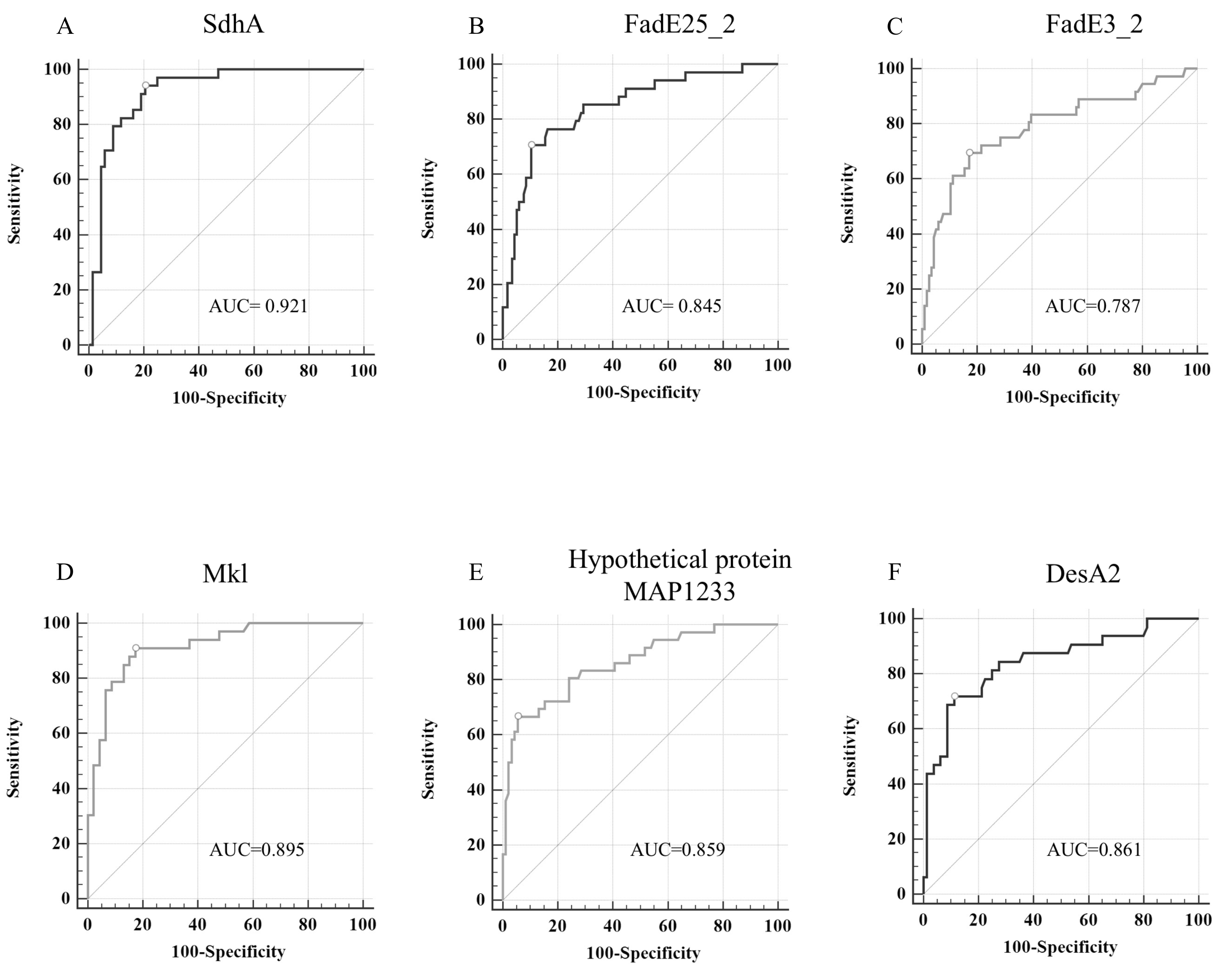

Supplement: Supplementary Figure 4 — Original image file for the immunoblot included in Figure 1. Immunoblot represents analysis of cell envelope proteins from MAP, M. avium subsp. hominisuis (MAH) and M. smegmatis (MS) extracted in two separate experiments (i.e., lanes 1–4 and 4–8) to confirm reproducibility. Lanes 1–4 were included in the cropped image in Figure 1. [file Image_4.JPEG]

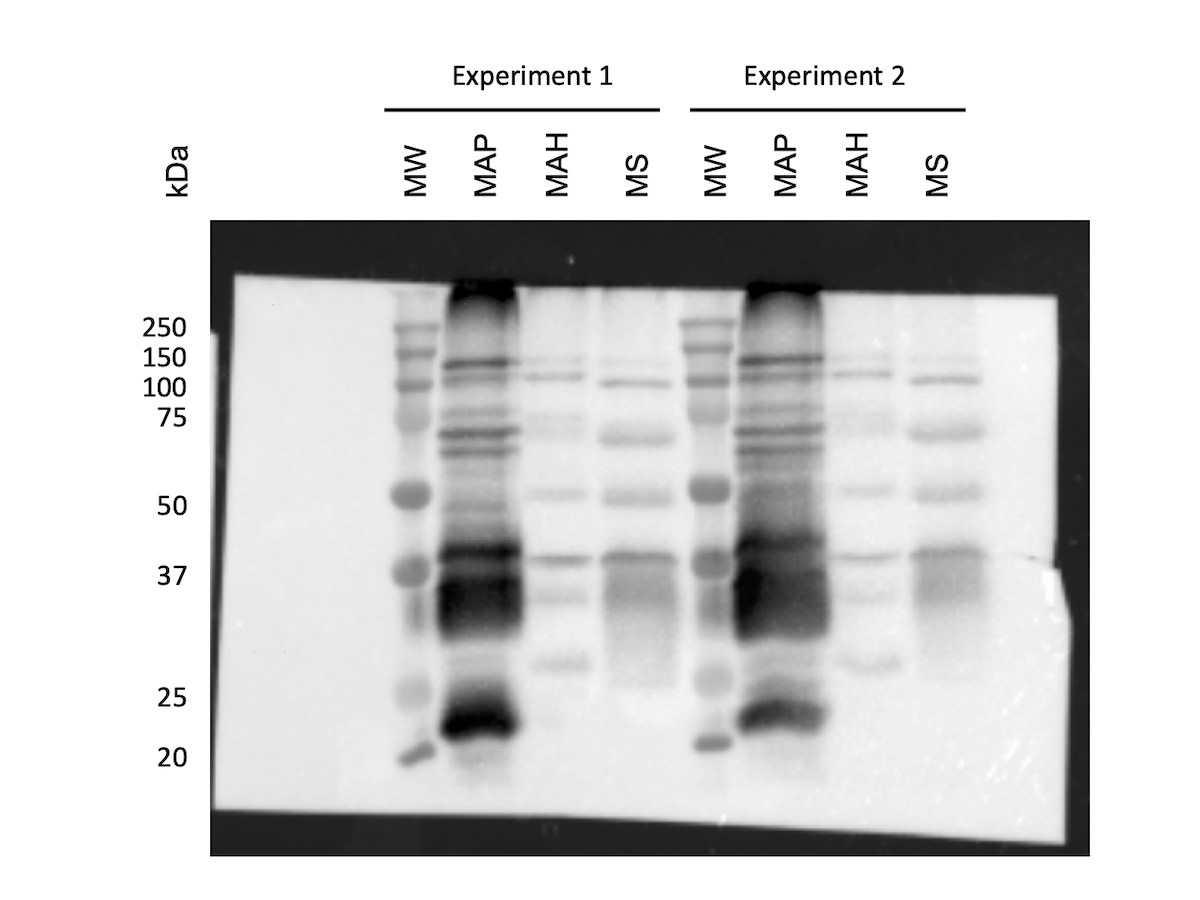

Supplement: Supplementary file 6 [file Image_5.TIFF]
